# Impact of disturbed diastolic vortex formation on viscous energy loss in the left ventricle: Quantitative 4D Flow MRI analysis of healthy controls and repaired atrioventricular septal defect patients

**DOI:** 10.1186/1532-429X-17-S1-P24

**Published:** 2015-02-03

**Authors:** Mohammed SM ElBaz, Emmeline Calkoen, Jos J Westenberg, Arno Roest, Rob J van der Geest

**Affiliations:** 1 Division of Image Processing, Radiology, Leiden University Medical Center (LUMC), Leiden, Netherlands; 2 Pediatric cardiology, Leiden University Medical Center, Leiden, Netherlands

## Background

Vortex formation in the left ventricle (LV) is suggested to contribute to efficient blood pumping and minimization of energy loss. Patients after atrioventricular septal defect (AVSD) repair may present abnormalities in valve morphology and subsequently develop altered LV inflow patterns [1], in which normal vortex formation may be disturbed. This may lead to energy loss. We aimed to analyze the association between disturbed vortex ring formation during diastole and viscous energy loss during diastole in AVSD-corrected patients compared to healthy controls.

## Methods

23 AVSD-repaired patients with NYHA class 1 and 2 (age: 20±8 years) and 23 age-matched healthy controls (age: 19±8 years) were included. All subjects (Table 1) underwent whole-heart 4D Flow MRI at 3T with free breathing, three-directional velocity-encoding of 150cm/s in all directions, spatial resolution 2.3×2.3×3.0-4.2mm^3^ and 30 retrospectively-gated phases reconstructed over one cardiac cycle. The LV cavity was manually segmented from the 4D Flow data. The Lambda2 method was used to derive the cores of 3D vortex ring structures from the velocity field inside the segmented LV cavity at the peak early (E) filling phase as described in [2]. Using Navier-Stokes energy equations, non-turbulent viscous energy loss (EL) was evaluated in the LV as the integration of viscous energy dissipation over diastolic period as described previously [3] with blood assumed as an incompressible Newtonian fluid. To get the EL per unit volume, EL was then normalized by the end diastolic volume. Inflow area was measured as previously described in [1]. Vortex Formation Time (VFT) was computed as described in [4] as VFT= (U*T)/D with U as the inflow velocity through the mitral orifice and D as the inflow diameter through the mitral orifice. Both U and D were averaged over the phases of early filling period. T is the time period of early filling. Measured parameters were compared using Wilcoxon rank sum test. Association between EL and VFT was evaluated using Pearson's correlation.

## Results

**Figure 1:**
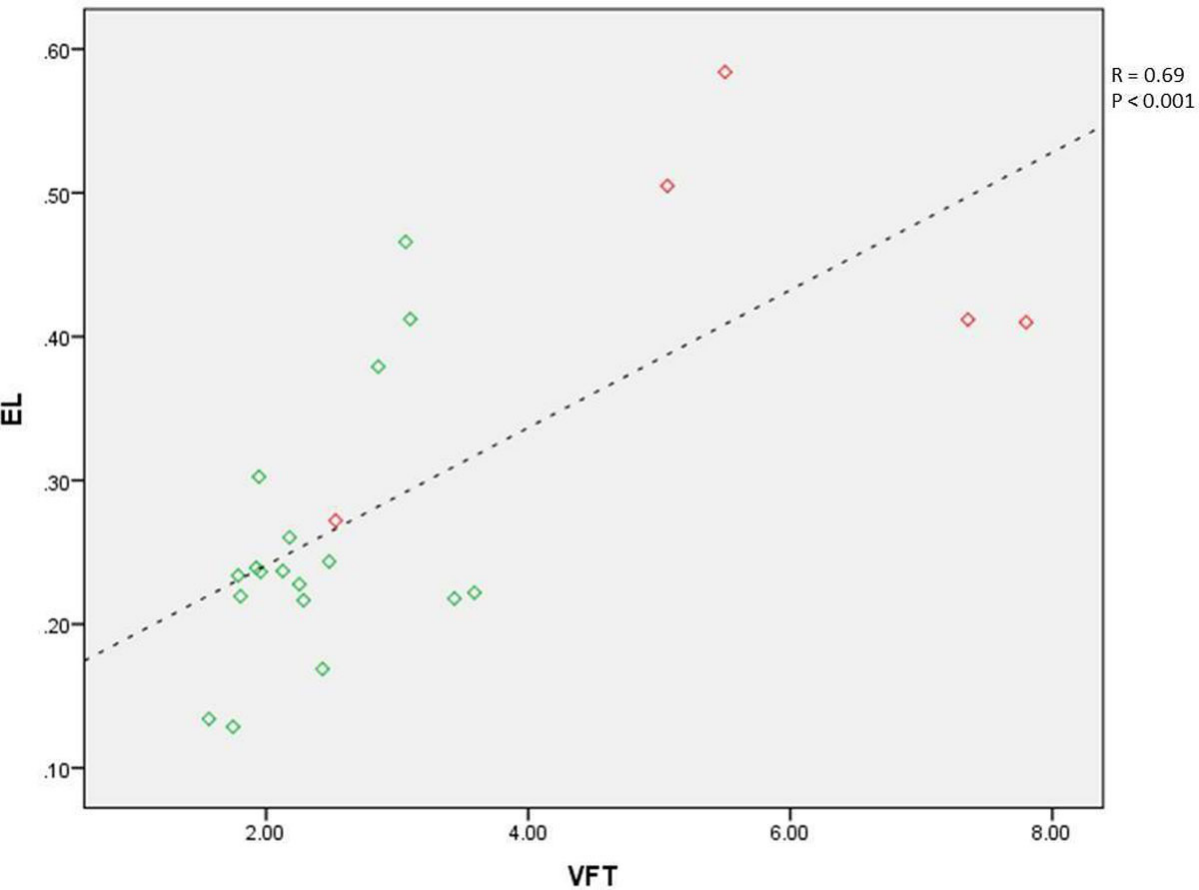
Correlation between vortex formation time (VFT) and viscous energy loss (EL in mJ) in patients, R=0.69. Patients with absent vortex ring core are marked in red. Dashed line corresponds to the regression line.

In all controls, a distinct vortex ring core was identified distal to the mitral valve at the E-peak. In 5 patients (22%) no vortex ring cores were identified. In these 5 patients, EL was significantly increased as compared to controls and their VFT was significantly higher than in controls. These patients showed a restricted inflow area compared to controls. In the remaining 18 patients, EL, VFT and inflow area were comparable to controls (Table 1). In patients, a good correlation was found between EL and VFT (R=0.69) (Figure 1).

**Table 1:** Viscous energy loss characteristics of AVSD- repaired patients in the presence and absence of E-filling vortex ring formation

	Control (N=23)	Patients with absent E-vortex ring core (N=5)	Patients with E-vortex ring core (N=18)
EL (mJ/mL) ^a^	0.21 ±0.07	0.77 ± 0.56*	0.25 ± 0.08

VFT	2.85 ±0.57	5.65 ± 2.1*	2.36 ± 0.61

Inflow area (cm2)	8.6 ± 1.9	5.7 ± 1.6*	9.08 ± 2.69

Age (yrs)	19 ± 8	18 ± 6	20 ± 8

Heart rate (bpm)	73 ± 13	82 ± 23	77 ± 10

Diastasis duration (ms)	91 ± 75	0 ± 0 (N=3) **	53 ± 68

E/A ratio	2.7 ± 0.7	2.9 ± 0.9 (N=3)**	2.4 ± 1.2

^a^ EL is normalized by end diastolic LV volume

*statistically significant with P<0.01 compared to controls

** Two cases had no A-wave

## Conclusions

Viscous energy loss is increased in the absence of diastolic vortex ring formation in AVSD-repaired patients with restricted inflow area. This is one of the first *in vivo* studies to quantitatively confirm the association between disturbed LV vortex formation and viscous energy loss from 4D Flow MRI.

## Funding

MSM ElBaz and J.J.M. Westenberg are financially supported by a grant from the Dutch Technology Foundation (STW). E.E. Calkoen is financially supported by a grant from the Willem-Alexander Kinder- en Jeugdfonds, M.S.
